# A robust transfer learning approach for high-dimensional linear regression to support integration of multi-source gene expression data

**DOI:** 10.1371/journal.pcbi.1012739

**Published:** 2025-01-10

**Authors:** Lulu Pan, Qian Gao, Kecheng Wei, Yongfu Yu, Guoyou Qin, Tong Wang

**Affiliations:** 1 Department of Biostatistics, School of Public Health, Fudan University, Shanghai, China; 2 Department of Health Statistics, School of Public Health, Shanxi Medical University, Taiyuan, China; 3 Key Laboratory of Coal Environmental Pathogenicity and Prevention (Shanxi Medical University), Ministry of Education, China; 4 Shanghai Institute of Infectious Disease and Biosecurity, Shanghai, China; 5 Key Laboratory of Public Health Safety of Ministry of Education, Key Laboratory for Health Technology Assessment, National Commission of Health, Fudan University, Shanghai, China; CANADA

## Abstract

Transfer learning aims to integrate useful information from multi-source datasets to improve the learning performance of target data. This can be effectively applied in genomics when we learn the gene associations in a target tissue, and data from other tissues can be integrated. However, heavy-tail distribution and outliers are common in genomics data, which poses challenges to the effectiveness of current transfer learning approaches. In this paper, we study the transfer learning problem under high-dimensional linear models with t-distributed error (Trans-PtLR), which aims to improve the estimation and prediction of target data by borrowing information from useful source data and offering robustness to accommodate complex data with heavy tails and outliers. In the oracle case with known transferable source datasets, a transfer learning algorithm based on penalized maximum likelihood and expectation-maximization algorithm is established. To avoid including non-informative sources, we propose to select the transferable sources based on cross-validation. Extensive simulation experiments as well as an application demonstrate that Trans-PtLR demonstrates robustness and better performance of estimation and prediction when heavy-tail and outliers exist compared to transfer learning for linear regression model with normal error distribution.

Data integration, Variable selection, T distribution, Expectation maximization algorithm, Genotype-Tissue Expression, Cross validation.

## Author summary

Many genetic loci have been shown to be associated with the mechanisms of important disease onset. Therefore, studying the expression of important genes contributes to the diagnosis and treatment of diseases. However, limited target gene expression data poses challenges to studying gene regulation. How to effectively integrate gene expression data from multiple sources is a key issue that needs to be addressed. In this study, we propose a robust transfer learning method aimed at improving the estimation and prediction performance of target gene expression data by integrating information from multiple data sources. By introducing a high-dimensional linear regression model with t-error distribution, our method addresses the shortcomings of previous transfer learning methods faced with heavy-tail distributions and outliers in genomics, providing robustness to complex data features. Extensive simulation experiments and an application demonstrate that our method exhibits better estimation and prediction performance when dealing with gene expression data with heavy-tail distributions and outliers.

## 1 Introduction

With the rapid development of genomic technologies, there are gene expression data available in public databases for many biological and medical research questions. Integrating multi-source data can overcome the problem of insufficient representativeness of target data, providing us with opportunities to explore the mechanisms of gene regulation and to understand the occurrence and progression of diseases [1,2]. However, due to the heterogeneity of biological samples from different sources, how to integrate the useful information from source data to improve estimation and prediction performance is a key challenge for the analysis and application of gene expression data [3,4].

### 1.1 Related work

To address the issue of systematic differences in multi-source gene expression data integration, current research mainly employs Meta-analysis (MA) and Data Merging (DM) approaches [5,6]. DM merges all samples in a unique dataset and subsequently performs analyses, while MA aggregates information from different samples at different stages of the analysis, for example, at the beginning by merging all samples or at the end by combining all the results of the separate analysis of specific samples [7,8]. Both methods increase the sample size by pooling all samples but fail to adjust for sample-specific heterogeneity, especially when focusing on the characteristics of specific target data. The heterogeneity among sources not only includes systematic variations caused by experimental conditions and measurement errors but also biological differences, such as variations in gene expression across different human tissue samples [9,10]. A unified model trained on all samples may exhibit bias and be unable to represent the true characteristics of the target sample of interest.

To address the challenge posed by such heterogeneity of source datasets and lack of representation of target data, transfer learning has emerged as a promising approach, aiming to transfer useful information from related but different source datasets to enhance the learning performance of the target data [11, 12]. When the target data is limited and the source data is large but biased, Bouveyron and Jacques (2010) proposed adaptive models to learn the target parameters through a linear transformation of the source model’s parameters, showing significant improvements over prediction from the target data only [13]. However, this approach gives very low adaption freedom due to the limiting of the number of transformation matrix elements learned from the target data to control the extent of transfer learning. Bastani (2021) proposed a more flexible two-step transfer learning estimator for high-dimensional linear models with a single informative auxiliary study [14]. By estimating the estimator from the source data and debiasing it using the target data, significant improvements in prediction performance for target data can be achieved. Furthermore, Li et al. (2022) further considered the estimation and prediction of a high-dimensional linear regression in the setting of two-step transfer learning with multiple source data and proposed a transferable source data detection algorithm to avoid negative transfer [15]. However, the transfer learning estimators proposed by Bastani (2021) and Li et al. (2022) both rely on quadratic optimization based on linear regression with normal distributed error, which is sensitive to heavy-tail and outliers that are common in practice [16, 17].

### 1.2 Our contributions

In this paper, building on the method proposed by Li et al. (2022) [15], we propose a transfer learning framework under high-dimensional linear models with *t*-distributed error, aimed at enhancing the robustness of transfer learning under conditions of heavy-tailed distributions and outliers, thereby improving the estimation and prediction of target data. The heavy-tailed characteristics of the t-distribution make it more robust to outliers compared to the normal distribution [18–22]. Different degrees of freedom parameters of the *t*-distribution can be estimated to adapt to source data with varying outliers or heavy-tailed error distributions [23]. By introducing *t*-distributed errors, we use the expectation-maximization (EM) algorithm to optimize the penalized likelihood function for estimating regression parameters. In the oracle case with known transferable source datasets, a three-step transfer learning algorithm is established, which combines the information from the target dataset and transferable source datasets, and adjusts their differences simultaneously. To avoid including non-informative sources, we propose to select transferable sources based on cross-validation, which ensures that the data integration will increase the prediction performance.

We not only achieve robustness improvements methodologically but demonstrate that our proposed method can transfer useful information from source datasets to target data and is robust to heavy tails and outliers through extensive simulations and application. Compared to other candidate methods, it exhibits higher accuracy in estimation, prediction, and variable selection.

## 2 Materials and methods

### 2.1 Penalized linear regression with *t*-distributed error

Regression analysis is one of the most widely used statistical methods to understand the relationship between a response and a set of predictors [24]. For subject *i* = 1,…, *n*, let *Y*_*i*_ be a response and $X_{i}={\left(X_{i}^{1},\ldots ,X_{i}^{p}\right)}^{T}$ be *p* predictors. The linear regression model can be written as

$$Y_{i}=X_{i}^{T}\beta +\varepsilon_{i},$$ (1)

where ***β*** = (*β*^1^,…, *β*^*p*^)^*T*^ is the coefficient vector of interest and *ε*_*i*_ is the random error. In this paper, we assume that the dimension *p* is high but ***β*** is sparse, that is, only a small subset of predictors is relevant to the response. In traditional linear regression, it is assumed that *ε*_*i*_ follows a normal distribution. However, in genomics data, this assumption may not always be met due to outliers or heavy-tailed distributions, then the ordinary least squares estimates may become biased or inefficient. In this paper, we consider the robust linear regression which assumes that *ε*_*i*_ follow a *t*-distribution, that is, *ε*_*i*_ ~ *t*(0, *σ*^2^, *ν*) with location 0, scale parameter *σ*^2^ and degrees of freedom *ν* [15]. The *t*-distribution has heavier tails than the normal distribution, making it less sensitive to outliers and more suitable for dealing with genomics data that exhibit non-normal errors [18].

The penalized log-likelihood function of ***θ*** = (***β***^*T*^, *σ*^2^, *ν*)^*T*^ for model (1) is given by $L\left(\beta ,\sigma^{2},\nu \right)-\lambda {\left\|\beta \right\|}_{1}$, where

$$L\left(\beta ,\sigma^{2},\nu \right)=\sum_{i=1}^{n}log\left\{t\left(Y_{i};X_{i}^{T}\beta ,\sigma^{2},\nu \right)\right\},$$

with

$$t\left(Y_{i};X_{i}^{T}\beta ,\sigma^{2},\nu \right)=\frac{\Gamma \left(\frac{\nu +1}{2}\right)}{\Gamma \left(\frac{\nu }{2}\right)\sqrt{\pi \nu \sigma^{2}}}{\left(1+\frac{{\left(Y_{i}-X_{i}^{T}\beta \right)}^{2}}{\nu \sigma^{2}}\right)}^{-\frac{\nu +1}{2}},$$ (2)

where Γ(∙) enotes the gamma function, *λ*‖***β***‖_1_ is the *l*_1_-penalty term for variable selection [25], and *λ* is a regularization parameter that determines the amount of shrinkage. The maximum likelihood estimator $\hat{\theta }$ is defined as the maximizer of $L\left(\beta ,\sigma^{2},\nu \right)-\lambda {\left\|\beta \right\|}_{1}$. However, directly optimizing the above objective function is computationally complex. In the following, we introduce an alternative representation of [2], which is based on the fact that the *t*-distribution can be written as a gamma-normal hierarchical form [26] as:

$$Y_{i}|\tau_{i}\sim N(X_{i}^{T}\beta ,\frac{\sigma^{2}}{\tau_{i}}),\tau_{i}\sim \Gamma \left(\frac{\nu }{2},\frac{\nu }{2}\right),i=1,\ldots ,n,$$ (3)

where *N*(∙) denotes the normal distribution and Γ(∙,∙) denotes the gamma distribution. The *τ*_*i*_ is a latent variable, then the gamma-normal hierarchical form leads to expectation-maximization (EM) algorithm [27] implementations for maximum likelihood estimation of the unknown parameters.

Given the data $D=\left(D_{1}^{T},\ldots ,D_{n}^{T}\right)$ with $D_{i}={\left(X_{i}^{T},Y_{i}\right)}^{T}$ for *i* = 1,…, *n*, and the latent variable vector ***τ*** = (*τ*_1_,…, *τ*_*n*_)^*T*^. According to the gamma-normal hierarchical form (3), the penalized log-likelihood function can be re-expressed as $L_{1}\left(\beta ,\sigma^{2};D,\tau \right)+L_{2}\left(\nu ;\tau \right)-\lambda {\left\|\beta \right\|}_{1}$, where

$$L_{1}\left(\beta ,\sigma^{2};D,\tau \right)=\sum_{i=1}^{n}\left\{log\frac{\sqrt{\tau_{i}}}{\sqrt{2\pi }\sigma }-\frac{\tau_{i}}{2\sigma^{2}}{\left(Y_{i}-X_{i}^{T}\beta \right)}^{2}\right\},$$

with

$$L_{2}\left(\nu ;\tau \right)=\sum_{i=1}^{n}log\frac{\tau_{i}^{\frac{\nu -2}{2}}e^{-\frac{\nu }{2}\tau_{i}}{\left(\frac{\nu }{2}\right)}^{\frac{\nu }{2}}}{\Gamma \left(\frac{\nu }{2}\right)}.$$ (4)

In the E-step, given the parameter values ***θ***^(*u*−1)^ at the (*u* − 1)-th iteration, the expectation of the complete log-likelihood is given as

$$Q\left(\theta ,\theta^{\left(u-1\right)}\right)=E\left\{L_{1}\left(\beta ,\sigma^{2};D,\tau \right)|D,\theta^{\left(u-1\right)}\right\}+E\left\{L_{2}\left(\nu ;\tau \right)|D,\theta^{\left(u-1\right)}\right\}-\lambda {\left\|\beta \right\|}_{1}.$$

In the M-step, the above objective function can be optimized by Newton-Raphson-type algorithm. The detailed procedure is presented in **Algorithm 1**.

**Algorithm 1** EM algorithm

**Input:** Data ***D*** and initial value ***θ***^(0)^.

At the *u*-th iteration, given ***θ***^(*u*−1)^ from the (*u* − 1)-th iteration:

 E-step. Compute the expectation $E\left\{L_{1}\left(\beta ,\sigma^{2};D,\tau \right)|D,\theta^{\left(u-1\right)}\right\}$ and $E\left\{L_{2}\left(\nu ;\tau \right)|D,\theta^{\left(u-1\right)}\right\}$.

 M-step. Update unknown parameters as ***θ***^(*u*)^ = argmax_***θ***_ *Q*(***θ*, *θ***^(*u*−1)^**)**.

Iterate E-step and M-step, until ‖***θ***^(*u*)^ − ***θ***^(*u*−1)^‖_1_ < 10^−6^.

**Output**: $\hat{\beta }=\beta^{\left(u\right)}$.

### 2.2 Target dataset, source dataset, and transferable dataset

Suppose there is a target dataset ${\left\{X_{i}^{\left(0\right)},Y_{i}^{\left(0\right)}\right\}}_{i\in I^{\left(0\right)}}$ and *S* independent source datasets ${\left\{{\left\{X_{i}^{\left(s\right)},Y_{i}^{\left(s\right)}\right\}}_{i\in I^{\left(s\right)}}\right\}}_{s=1,\ldots ,S}$, where $I^{\left(0\right)}$ is the index set of subjects belonging to target dataset with sample size *n*_0_, and $I^{\left(s\right)}$ (*s* = 1,…, *S*) is the index set of subjects belonging to the *s*-th source dataset with sample size *n*_*s*_. The regression models corresponding to the target dataset and the source datasets are:

$$Y_{i}^{\left(s\right)}=X_{i}^{T\left(s\right)}\beta^{\left(s\right)}+\varepsilon_{i}^{\left(s\right)},s=0,\ldots ,S,i\in I^{\left(s\right)}.$$ (5)

where ***β***^(0)^ is the coefficient of the target dataset, and ***β***^(*s*)^ (*s* = 1,…, *S*) is the coefficient of the *s*-th source dataset.

Our goal is to transfer useful information from the source datasets to the target dataset, to improve the estimation accuracy of ***β***^(0)^. The index set of the transferable source datasets is denoted as T, which is a subset of {1,…, *S*}. Intuitively, the closer ***β***^(*s*)^ of the *s*-th source dataset is to ***β***^(0)^ of the target dataset, the more transferable that source dataset may be. Deeply, if we combine the *s*-th source dataset with the target dataset, the estimation or prediction performance is improved relative to using the target dataset alone, then we consider that source dataset to be transferable.

### 2.3 Transfer learning with known transferable dataset

We first consider that index set T for the transferable source dataset is known. Motivated by Li et al. (2023) [28], we propose a three-step transfer learning framework for linear regression model [5]. In the first step, we fit regression model using Algorithm 1 in each transferable source dataset s∈T, and obtain ${\hat{\beta }}^{\left(s\right)}$. In the second step, we use the target data to measure the difference between ***β***^(*s*)^ (s∈T) and ***β***^(0)^, which is denoted as ${\hat{\delta }}^{\left(s\right)}$. In the third step, we combine the target dataset and transferable source datasets to jointly estimate ***β***^(*TL*)^, and use ${\hat{\delta }}^{\left(s\right)}$ to adjust the differences. The details of the algorithm are shown in **Algorithm 2**, in which the regularization parameters $\lambda_{\beta }^{\left(s\right)}(s\in T)$, *λ*_*δ*_, and *λ*_*β*_ are selected by cross validation.

**Algorithm 2** Transfer learning with known transferable dataset T

**Input:** Target dataset ${\left\{D_{i}={\left(X_{i}^{T},Y_{i}\right)}^{T}\right\}}_{i\in I^{\left(0\right)}}$, source datasets ${\left\{D_{i}={\left(X_{i}^{T},Y_{i}\right)}^{T}\right\}}_{i\in I^{\left(s\right)}}$ for s∈T, and index set T for the transferable dataset.

Step 1. Fit the regression model in each transferable source dataset. For s∈T, compute

$${\hat{\beta }}^{\left(s\right)}=\underset{\beta }{\text{argmax}}\left\{L\left(\beta ;{\left\{D_{i}\right\}}_{i\in I^{\left(s\right)}}\right)-\lambda_{\beta }^{\left(s\right)}{\left\|\beta \right\|}_{1}\right\}.$$

Step 2. Measure the differences using target dataset. For s∈T, compute

$${\hat{\delta }}^{\left(s\right)}=\underset{\delta }{\text{argmax}}\left\{L\left({\hat{\beta }}^{\left(s\right)}+\delta ;{\left\{D_{i}\right\}}_{i\in I^{\left(0\right)}}\right)-\lambda_{\delta }^{\left(s\right)}{\left\|\delta \right\|}_{1}\right\}.$$

Step 3: Joint estimation using target dataset and transferable source datasets, and adjusting the differences. Compute

$${\hat{\beta }}^{\left(TL\right)}=\underset{\beta }{\text{argmax}}\left\{L\left(\beta ;{\left\{D_{i}\right\}}_{i\in I^{\left(0\right)}}\right)+\sum_{s\in T}L\left(\beta -{\hat{\delta }}^{\left(s\right)};{\left\{D_{i}\right\}}_{i\in I^{\left(s\right)}}\right)-\lambda_{\beta }{\left\|\beta \right\|}_{1}\right\}.$$

The above optimization problems can be achieved by running EM **Algorithm 1**.

**Output:** ${\hat{\beta }}^{\left(TL\right)}$.

### 2.4 Detecting the transferable dataset

We now consider how to detect the index set T for the transferable dataset. As previously mentioned, if we combine the *s*-th source dataset with the target dataset, the estimation or prediction performance is improved relative to using the target dataset alone, then we consider that source dataset to be transferable. Inspired by Eaton et al. (2008) and Zhang and Zhu (2022) [29, 30], we propose a cross-validation framework for detecting the transferable dataset. We randomly split the subjects in target dataset into two disjoint equal-size groups $I^{\left(0\right)}=I^{\left(0,tr\right)}⋃I^{\left(0,va\right)}$, where $I^{\left(0,tr\right)}$ is the training set and $I^{\left(0,va\right)}$ is the validation set. We first fit regression model using Algorithm 1 in training set $I^{\left(0,tr\right)}$, and obtain ${\hat{\beta }}^{\left(0\right)}$. For a given *s* = 1,…, *S*, then we perform transfer learning using Algorithm 1 in $I^{\left(0,tr\right)}$ with the *s*-th source dataset, and obtain ${\hat{\beta }}^{\left(s\right)}$. We evaluate the performance of ${\hat{\beta }}^{\left(0\right)}$ and ${\hat{\beta }}^{\left(s\right)}$ on the validation set, by computing ${\hat{T}}^{\left(s\right)}=L\left({\hat{\beta }}^{\left(0\right)};{\left\{D_{i}\right\}}_{i\in I^{\left(0,va\right)}}\right)-L\left({\hat{\beta }}^{\left(s\right)};{\left\{D_{i}\right\}}_{i\in I^{\left(0,va\right)}}\right)$. The ${\hat{T}}^{\left(s\right)}<0$ indicates that the transfer of the *s*-th source dataset makes the prediction performance increase, so we consider s∈T. If ${\hat{T}}^{\left(s\right)}>0$ but not exceeding $t\left(L\left({\hat{\beta }}^{\left(0\right)};{\left\{D_{i}\right\}}_{i\in I^{\left(0,va\right)}}\right)\vee 0.01\right)$ for a given threshold t, we also consider s∈T. The details of the algorithm are shown in **Algorithm 3**.

**Algorithm 3** Transfer learning with unknown transferable dataset T

**Input**: Target dataset ${\left\{D_{i}={\left(X_{i}^{T},Y_{i}\right)}^{T}\right\}}_{i\in I^{\left(0\right)}}$, source dataset ${\left\{D_{i}={\left(X_{i}^{T},Y_{i}\right)}^{T}\right\}}_{i\in {⋃}_{s=1}^{S}I^{\left(s\right)}}$.

1. Detecting transferable dataset

Randomly split $I^{\left(0\right)}$ into two disjoint equal-size groups $I^{\left(0\right)}=I^{\left(0,tr\right)}⋃I^{\left(0,va\right)}$.

 (1) Compute ${\hat{\beta }}^{\left(0\right)}=\underset{\beta }{\text{argmax}}\left\{L\left(\beta ;{\left\{D_{i}\right\}}_{i\in I^{\left(0,tr\right)}}\right)-\lambda_{\beta }^{\left(0\right)}{\left\|\beta \right\|}_{1}\right\}$.

 (2) For *s* = 1,…, *S*, compute ${\hat{\beta }}^{\left(s\right)}$ by running the **Algorithm 2** with ${\left\{D_{i}\right\}}_{i\in I^{\left(0,tr\right)}\cup I^{\left(s\right)}}$.

 (3) For *s* = 1,…, *S*, compute ${\hat{T}}^{\left(s\right)}=L\left({\hat{\beta }}^{\left(0\right)};{\left\{D_{i}\right\}}_{i\in I^{\left(0,va\right)}}\right)-L\left({\hat{\beta }}^{\left(s\right)};{\left\{D_{i}\right\}}_{i\in I^{\left(0,va\right)}}\right)$.

Let $\hat{T}=\left\{s:{\hat{T}}^{\left(s\right)}<t\left(L\left({\hat{\beta }}^{\left(0\right)};{\left\{D_{i}\right\}}_{i\in I^{\left(0,va\right)}}\right)\vee 0.01\right)\right\}$.

2. Run **Algorithm 2** with target dataset ${\left\{D_{i}\right\}}_{i\in I^{\left(0\right)}}$, and detected transferable source dataset ${\left\{D_{i}\right\}}_{i\in I^{\left(s\right)}}$ for $s\in \hat{T}$ to obtain ${\hat{\beta }}^{\left(TL\right)}$.

**Output**: ${\hat{\beta }}^{\left(TL\right)}$

## 3. Numerical experiments

In this section, we conduct simulation studies to evaluate the performance of the proposed method. We compare our proposed transfer learning framework for penalized *t*-linear regression (Trans-PtLR based on Algorithm 3) with (i) Naive Trans-PtLR: based on Algorithm 2 using all source datasets; (ii) PtLR: based on Algorithm 1 using only target dataset. We also compare them with the following three methods, Trans-PNLR, Naive Trans-PNLR, and PNLR, which consider the penalized normal-linear regression.

### 3.1 Simulation

We consider a target dataset with *n*_0_ = 150, and *S* = 10 source datasets with *n*_1_ = … *n_S_* = 100. For *s* = 0,…, *S*, we set *p* = 500 and the predictors $X_{i}^{\left(s\right)}$ is drawn from the multivariate normal distribution with mean zero and covariance matrix **Σ**(*ρ*), where **Σ**(*ρ*) is the first-order autoregressive correlation structure with *ρ* = 0.7. The response is generated as

$$Y_{i}^{\left(s\right)}=X_{i}^{T\left(s\right)}\beta^{\left(s\right)}+\varepsilon_{i}^{\left(s\right)}.$$

For the coefficient ***β***^(0)^ = (*β*^1(0)^,…, *β*^*p*(0)^)^*T*^ corresponding to target dataset, we set *k* = 16, *β*^*j*(0)^ = 0.5 for *j* = 1,…, *k* and *β*^*j*(0)^ = 0 otherwise. We randomly select $\left|T\right|\in \{0,2,4,6,8,10\}$ source datasets as the transferable datasets with the corresponding coefficients set to be

$$\beta^{j\left(s\right)}=\left\{\begin{array}{cc} \beta^{j\left(0\right)}-\frac{h}{100}I\{j\in H^{\left(s\right)}\} & s\in T,\\ \frac{h}{100}I\{j\in H^{\left(s\right)}\cup [k]\} & s\notin T, \end{array}\right.$$

where *H*^(*s*)^ is a random subset of {17,…, *p*} with |*H*^(*s*)^| = 50 and *h* ∈ {10,20} controls the heterogeneity levels between target dataset and source datasets.

To evaluate the robustness of the proposed method, we consider four distributions of the error. For *s* = 0,…, *S*, we set (i) normal distribution as *ε*^(*s*)^ ~ *N*(0, 1); (ii) *t*-distribution as *ε*^(*s*)^ ~ *t*(0, 1, 5); (iii) contaminated normal distribution as *ε*^(*s*)^ ~ 0.9*N*(0, 1) + 0.1*N*(0, 10), which generates outliers; (iv) skew *t*-distribution as *ε*^(*s*)^ ~ *St*(0, 1, 5, skewness = 1).

We conduct 100 replications. In each replication, to evaluate the estimation accuracy, we calculate the estimation error ${\left\|{\hat{\beta }}^{\left(0\right)}-\beta^{\left(0\right)}\right\|}_{2}^{2}$; to evaluate the prediction accuracy, we randomly split the target dataset into five folds, using four folds to train and the remaining fold to calculate the mean prediction error ${\left({\hat{Y}}_{i}-Y_{i}\right)}^{2}$ with ${\hat{Y}}_{i}=X_{i}^{T\left(0\right)}{\hat{\beta }}^{\left(0\right)}$; to evaluate the accuracy of the variable selection, we calculate the proportion of true predictors among predictors selected by *l*_1_-regularization (defined as precision) and the proportion of selected true predictors among all true predictors (defined as recall).

The threshold t in **Algorithm 3** is set to be 0.1. Results corresponding to t=0.05 or 0.15 are available in S1–S4 Fig, which shows that the estimates are insensitive to a small range of t.

### 3.2 Application to the prediction of gene expression

Gene expression data can be used to explore the gene-gene interaction and gene regulation for a better understanding of molecular processes of pathogenesis and the purpose of prediction of disease. For instance, discovering gene expression patterns that are characteristic of a certain disease to differentiate between healthy and diseased individuals.

In this section, we consider Primary Familial Brain Calcification (PFBC) which is a hereditary neurodegenerative disease characterized by progressive bilateral cerebral calcifications, accompanied by various symptoms such as dystonia, ataxia, Parkinson’s disease, dementia, depression, headaches, and epilepsy [31]. Currently, the exact etiology of PFBC remains unclear. Recent studies indicate that variations in the JAM2 gene result in decreased JAM2 mRNA expression and loss of JAM2 protein in patient fibroblasts, consistent with the loss-of-function mechanism [32, 33]. JAM2 is a protein-coding gene located on chromosome 21, and the encoded protein is a cell adhesion molecule expressed in various tissues and cell types, influencing cell migration, adhesion, and interactions [34]. The expression pattern of JAM2 may be regulated under physiological and pathological conditions. Therefore, obtaining the expression levels of JAM2 is of great significance for the diagnosis of PFBC. Given the technical challenges and ethical concerns associated with brain tissue sequencing, constructing model to predict the expression levels of JAM2 in target brain tissues holds potential clinical diagnostic value for PFBC. The objective of this application is to train a prediction model of JAM2 expression levels in target brain tissues, transferring useful information from other tissues or cell types to improve the prediction of JAM2 expression levels in the target brain tissue.

We evaluate the predictive performance of our proposed algorithm using tissue-specific gene expression data from the Genotype-Tissue Expression (GTEx) version 8 dataset, which consists of 838 donors and 17,382 samples from 54 non-diseased tissue types (S1 Table). In the analysis of this study, we used 49 tissues or cell lines that had at least 70 individuals, including a total of 17,329 samples from 838 donors. Based on previously published literature and publicly available lists of central nervous system (CNS)-related genes, the CNS-related genes were assembled as MODULE 137, including 546 genes as well as 1,632 additional genes that are significantly enriched in the same experiments as the genes of the module. These genes may participate in similar biological pathways or regulatory networks, working together in the central nervous system to perform specific functions. Therefore, they provide an important foundation for our study of JAM2 genes in PFBC. We consider 13 brain tissues as target tissues and the remaining 36 tissues as source tissues. The average sample size of target tissues and source tissues was 203 and 408, respectively. After excluding missing values, the final predictors included 1292 genes.

To compare the performance of the proposed method, we conduct Trans-PNLR and Trans-PtLR models in each tissue based on the association of the JAM2 gene and other CNS-related genes and identify the informative tissues to transfer to improve the performance of the target model in each brain tissue. We also conduct Naive Trans-PNLR and Naive Trans-PtLR to understand the total information level of all source tissues. We also conduct PNLR and PtLR to evaluate the prediction performance using only the target tissue, without transforming information from other source tissues. The response variable is the JAM2 expression level and covariates are the expression levels of other genes included in MODULE 137. For each model, we randomly split the target dataset into five folds, using four folds to train and the remaining fold to calculate the mean prediction error ${\left({\hat{Y}}_{i}-Y_{i}\right)}^{2}$ with ${\hat{Y}}_{i}=X_{i}^{T\left(0\right)}{\hat{\beta }}^{\left(0\right)}$. The data is standardized before analysis.

## 4 Results

### 4.1 Simulation results

**Fig 1:**
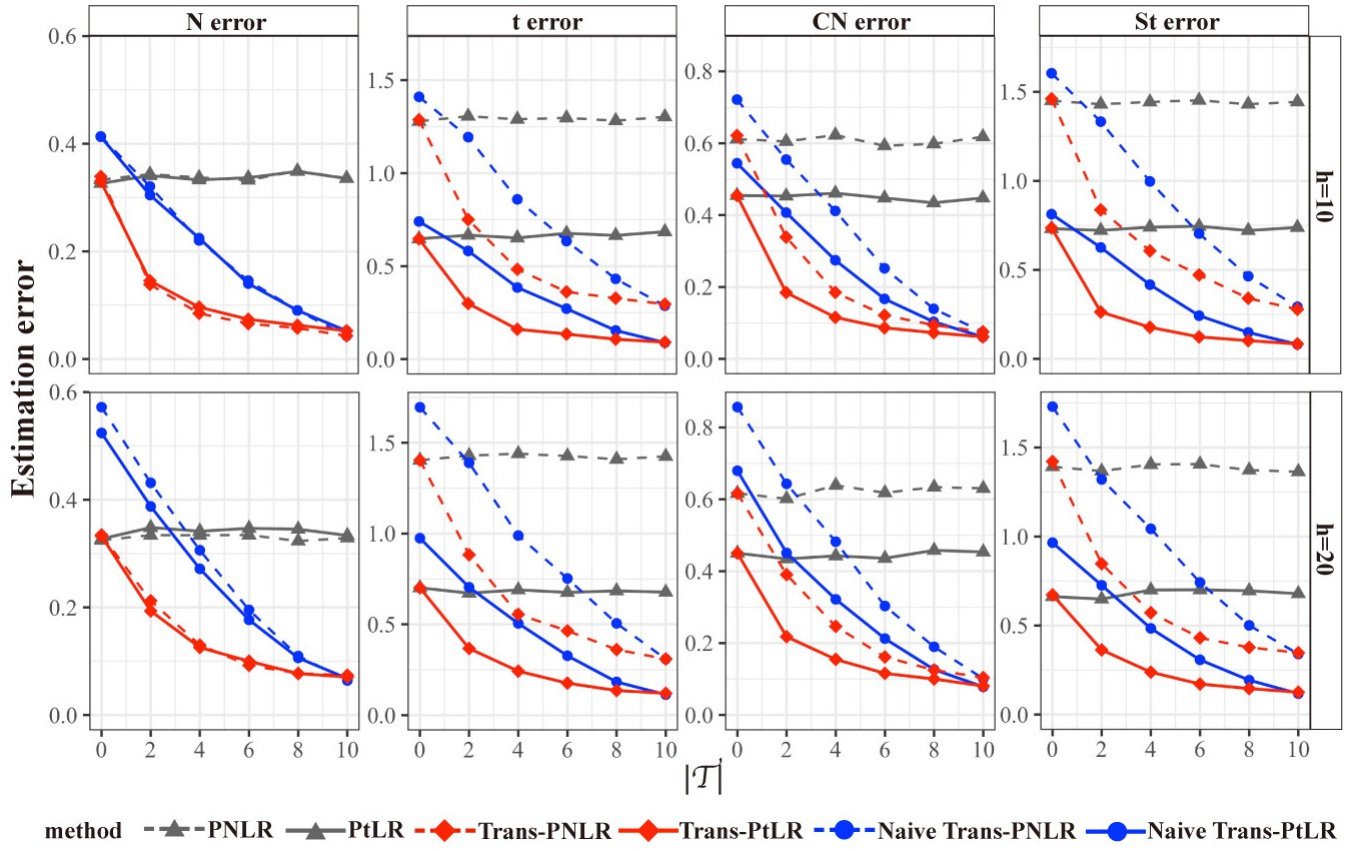
Estimation error ${\left\|{\hat{\beta }}^{\left(0\right)}-\beta^{\left(0\right)}\right\|}_{2}^{2}$ of candidate methods with a threshold t of 0.1. The estimation error is evaluated under different heterogeneity levels (*h* = 10 or 20), different numbers of transferable source datasets ($\left|T\right|\in \{0,2,4,6,8,10\}$), and different distributions of the error (*ε*^(*s*)^ follows normal distribution (N), *t*-distribution (t), contaminated normal distribution (CN), and skew *t*-distribution (St)).

**Fig 2:**
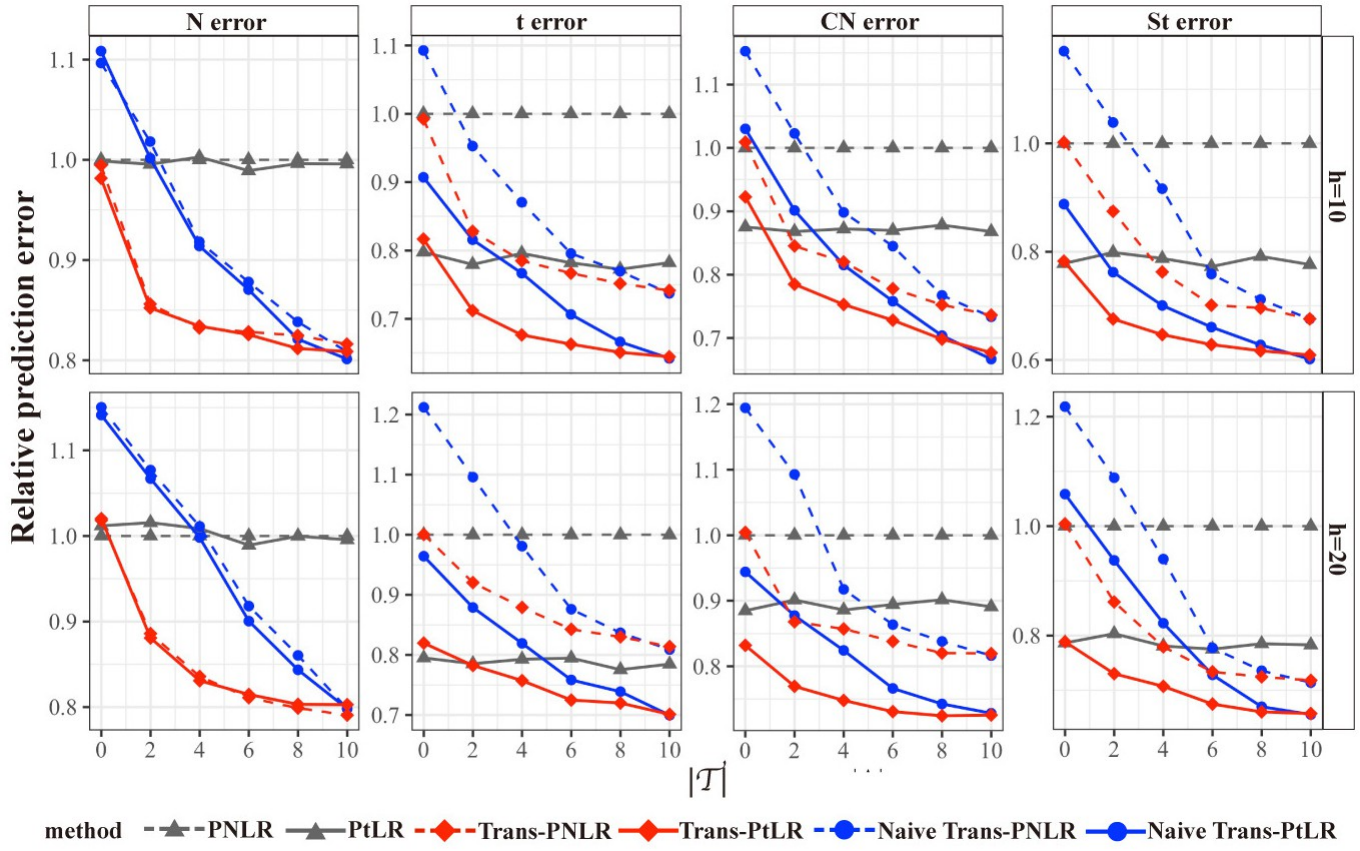
Relative prediction error of candidate methods relative to the PNLR with a threshold t of 0.1. The relative prediction error is evaluated under different heterogeneity levels (*h* = 10 or 20), different numbers of transferable source datasets ($\left|T\right|\in \{0,2,4,6,8,10\}$), and different distributions of the error (*ε*^(*s*)^ follows normal distribution (N), *t*-distribution (t), contaminated normal distribution (CN), and skew *t*-distribution (St)). We randomly split the target dataset into five folds, using four folds to train and the remaining fold to calculate the mean prediction error ${\left({\hat{Y}}_{i}-Y_{i}\right)}^{2}$ with ${\hat{Y}}_{i}=X_{i}^{T\left(0\right)}{\hat{\beta }}^{\left(0\right)}$.

Figs 1 and 2 and Table 1 show the estimation error, relative prediction error, and variable selection precision and recall of candidate methods under different heterogeneity levels (*h* = 10 or 20), different numbers of transferable source datasets ($\left|T\right|\in \{0,2,4,6,8,10\}$), and different distributions of the error (*ε*^(*s*)^ follows normal distribution (N), *t*-distribution (t), contaminated normal distribution (CN), and skew *t*-distribution (St)).

**Table 1:** Precision and recall (in parentheses) of candidate methods under different heterogeneity levels (*h* = 10 or 20), different numbers of transferable source datasets ($\left|T\right|\in \{0,2,4,6,8,10\}$), and different distributions of the error (*ε*^(*s*)^ follows normal distribution (N), *t*-distribution (t), contaminated normal distribution (CN), and skew *t*-distribution (St)).

	N error		t error		CN error		St error	
	PNLR	PtLR	PNLR	PtLR	PNLR	PtLR	PNLR	PtLR
*h* = 10								
No transfer	0.71 (1.00)	0.72 (1.00)	0.71 (0.92)	0.73 (0.99)	0.71 (0.99)	0.72 (1.00)	0.71 (0.92)	0.74 (0.99)
Transfer								
$\left|T\right|=0$	0.69 (1.00)	0.71 (1.00)	0.68 (0.93)	0.72 (0.99)	0.67 (0.99)	0.69 (1.00)	0.68 (0.93)	0.72 (0.98)
$\left|T\right|=2$	0.76 (1.00)	0.86 (1.00)	0.65 (0.99)	0.72 (1.00)	0.62 (1.00)	0.77 (1.00)	0.65 (0.99)	0.73 (1.00)
$\left|T\right|=4$	0.82 (1.00)	0.95 (1.00)	0.64 (1.00)	0.78 (1.00)	0.67 (1.00)	0.89 (1.00)	0.65 (1.00)	0.75 (1.00)
$\left|T\right|=6$	0.90 (1.00)	0.97 (1.00)	0.67 (1.00)	0.86 (1.00)	0.75 (1.00)	0.96 (1.00)	0.69 (1.00)	0.85 (1.00)
$\left|T\right|=8$	0.94 (1.00)	0.99 (1.00)	0.74 (1.00)	0.92 (1.00)	0.82 (1.00)	0.97 (1.00)	0.71 (1.00)	0.91 (1.00)
$\left|T\right|=10$	0.96 (1.00)	0.99 (1.00)	0.78 (1.00)	0.95 (1.00)	0.88 (1.00)	0.97 (1.00)	0.75 (1.00)	0.95 (1.00)
*h* = 20								
No transfer	0.71 (1.00)	0.72 (1.00)	0.71 (0.91)	0.73 (0.99)	0.71 (0.99)	0.71 (1.00)	0.70 (0.90)	0.73 (0.99)
Transfer								
$\left|T\right|=0$	0.70 (1.00)	0.72 (1.00)	0.68 (0.92)	0.73 (0.98)	0.70 (0.99)	0.72 (1.00)	0.68 (0.91)	0.71 (0.99)
$\left|T\right|=2$	0.65 (1.00)	0.79 (1.00)	0.64 (0.99)	0.76 (1.00)	0.68 (1.00)	0.76 (1.00)	0.65 (0.98)	0.68 (1.00)
$\left|T\right|=4$	0.73 (1.00)	0.85 (1.00)	0.63 (0.99)	0.79 (1.00)	0.66 (1.00)	0.81 (1.00)	0.68 (1.00)	0.77 (1.00)
$\left|T\right|=6$	0.78 (1.00)	0.92 (1.00)	0.63 (1.00)	0.86 (1.00)	0.70 (1.00)	0.85 (1.00)	0.69 (1.00)	0.85 (1.00)
$\left|T\right|=8$	0.82 (1.00)	0.96 (1.00)	0.68 (1.00)	0.84 (1.00)	0.73 (1.00)	0.91 (1.00)	0.72 (1.00)	0.90 (1.00)
$\left|T\right|=10$	0.86 (1.00)	0.97 (1.00)	0.66 (1.00)	0.89 (1.00)	0.74 (1.00)	0.94 (1.00)	0.74 (1.00)	0.93 (1.00)

Precision: the proportion of true predictors among predictors selected by *l*_1_-regularization. Recall (in parentheses): the proportion of selected true predictors among all true predictors.

For the normal distributed (N) error, Trans-PNLR and Trans-PtLR perform better than PNLR and PtLR, which indicates that transfer learning can help us extract additional information from the source datasets to improve the accuracy of estimation and prediction on the target dataset. As the numbers of transferable datasets $\left|T\right|$ increases, the accuracy of estimation and prediction becomes higher. Trans-PNLR and Trans-PtLR also perform better than Naive Trans-PNLR and Naive Trans-PtLR, especially when $\left|T\right|$ is relatively small. The probable reason is that when the number of transferable source datasets is small, transferring all the source datasets will result in negative transfer. Therefore, it is necessary to adopt the Algorithm 3 to detect the transferable dataset (S2 Table). We also observe that when the heterogeneity levels between target dataset and source datasets are smaller (*h* = 10), the accuracy of estimation and prediction for Trans-PNLR and Trans-PtLR is higher.

For the comparison between Trans-PNLR and Trans-PtLR, when the error is generated from normal distribution that contains no outliers, the performances of these two methods are quite similar. When the error is generated from *t*-distribution with heavy tail, Trans-PtLR has a smaller estimation error and prediction error than Trans-PNLR. When the error is generated from contaminated normal distributions, Trans-PtLR is less sensitive to potential outliers than Trans-PNLR. When the error is generated from skewed *t*-distribution which is skewed and heavy-tailed, Trans-PtLR also achieved better estimation and prediction performance than Trans-PNLR. As for genomics data, outliers and non-normal errors are likely to be encountered, so our proposed Trans-PtLR may be more suitable for application. For single regression coefficients with non-zero true effect, the estimation error exhibits the similar trend as Fig 1, while for regression coefficients with zero true effect, the estimation errors approach zero due to being compressed to zero by the *l*_1_-penalty (see S5 and S6 Figs). We also explored the impact of estimation performance when sample size of target and sources vary. After correctly identifying transferable datasets, even if the sample size of the sources is small, the estimation performance will still not be worse than using only the target data (S7 Fig).

We can find from Table 1 that the recall of Trans-PtLR and Trans-PNLR is close to 1, but in some scenarios, such as when *ε*^(*s*)^ follows a *t*-distribution (t) or skew *t*-distribution (St), for *h* = 10 or 20 and $\left|T\right|=0$, the recall of Trans-PtLR is higher than that of Trans-PNLR. This indicates that our method almost never misses important predictors. We also found that in most cases, the Precision of Trans-PtLR is higher than that of Trans-PNLR. This suggests that while Trans-PNLR may select more irrelevant variables, Trans-PtLR more accurately excludes unimportant predictors, thereby enhancing precision.

### 4.2 Predicting the gene expression

**Fig 3:**
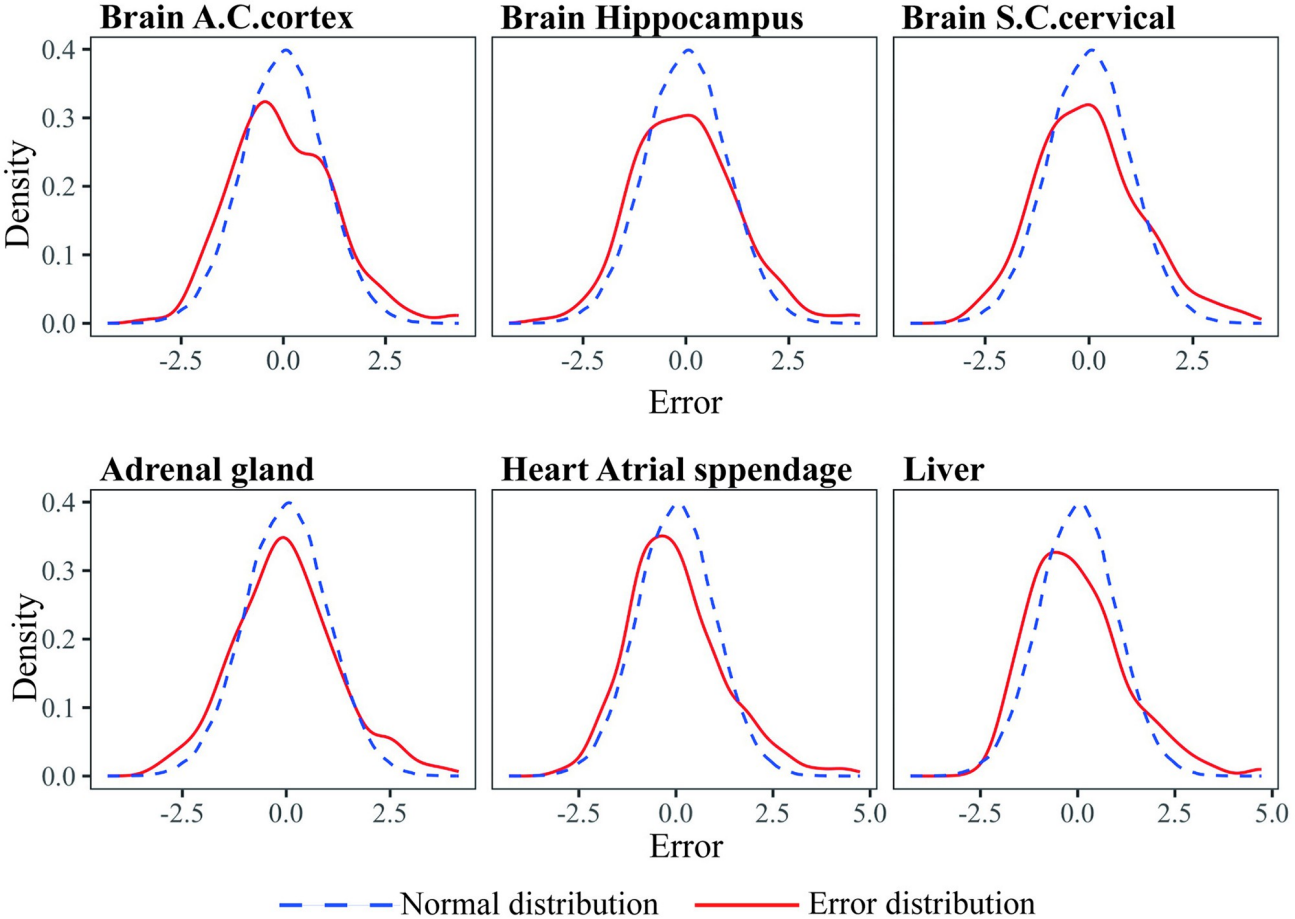
Distribution of error by normal linear regression for six tissues compared with normal distribution.

Before the analysis, we first fit a normal linear regression model for all tissues and conducted model diagnoses, then, we took several target tissues and source tissues as examples, plotted the distribution of error and compared it to the normal distribution. As shown in Fig 3, the distribution of error is heavy-tailed or may contain outliers, indicating that fitting the tissue-specific gene expression data with a normal linear regression may not be appropriate. It is reasonable to assume the error term following t distributions to reduce the effects of the potential outliers. The transferable sources detected by all methods and the sample size of all transferable sources are available in S3 and S4 Tables.

**Fig 4:**
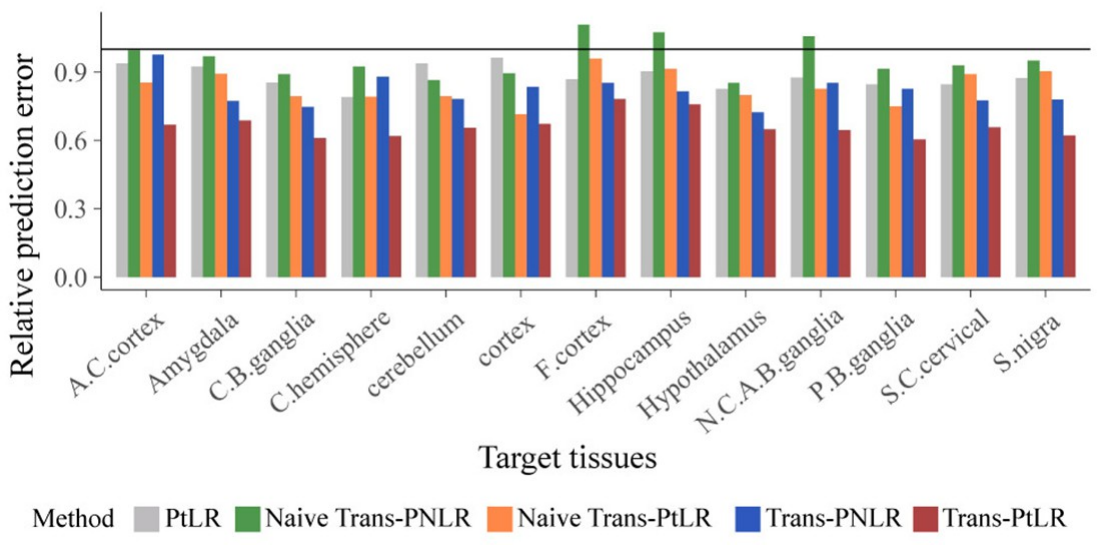
Relative prediction error of PtLR, Naive Trans-PNLR, Naive Trans-PtLR, Trans-PNLR, and Trans-PtLR relative to the PNLR. We randomly split the data in target tissues into five folds, using four folds to train and the remaining fold to calculate the mean prediction error ${\left({\hat{Y}}_{i}-Y_{i}\right)}^{2}$ with ${\hat{Y}}_{i}=X_{i}^{T\left(0\right)}{\hat{\beta }}^{\left(0\right)}$.

Fig 4 shows the relative prediction error of five models (PtLR, Naive Trans-PNLR, Naive Trans-PtLR, Trans-PNLR, and Trans-PtLR) relative to the PNLR using CNS gene expression levels to predict JAM2 gene expression levels in 13 brain tissues. The results show that transfer learning methods significantly reduce prediction errors in most scenarios. This highlights the practical advantages of integrating useful information from source tissues.

It is noteworthy that the model’s performance is poorer when transferable source datasets are not pre-identified (as in Naive Trans PNLR and Naive Trans PtLR). This emphasizes the importance of using the proposed transferable source detection algorithm, which effectively identifies valuable source information and improves predictive performance, demonstrating its practical applicability in complex data integration tasks.

Furthermore, the results suggest that transfer learning within the *t*-linear regression (Trans-PtLR) framework outperforms the normal regression (Trans-PNLR) framework. Transfer learning through Trans-PNLR reduced the average prediction error by 18.3%, while Trans-PtLR further reduced this by 33.6% (with Trans-PtLR averaging a 15.3% reduction in prediction error compared to Trans-PNLR). The Wilcoxon signed-rank test shows that the difference in prediction errors reduced by these two methods has a *P*-value of 0.0002. This significant improvement indicates that modeling with heavy-tailed t-distributed errors can better handle outliers and enhance the robustness of real-world datasets, particularly in gene expression data where heavy tails and noise are common.

## 5 Discussion

This paper studies robust transfer learning for penalized *t*-linear regression model under high-dimensional data and its application in gene expression prediction. We conduct a three-step transfer learning algorithm to obtain the joint estimator and use a data-driven transferable source detection algorithm to prevent negative transfer. Extensive simulations demonstrate the better performance and robustness of estimation and prediction compared with transfer learning for normal linear regression when outliers or heavy-tailed errors exist. In this application based on GTEx data, we build an expression prediction model for the JAM2 gene which treats 1292 other genes as predictors. We consider 13 brain tissues as target tissues and the remaining 36 tissues as source tissues. Our proposed method also shows higher accuracy for prediction compared to another candidate approach in each target tissue.

One of the key distinctions between our Trans-PtLR method and existing methods Trans-PNLR lies in the handling of error distributions. Unlike Trans-PNLR, which relies on the assumption of normal distribution, our method incorporates *t*-distributed errors, making it more robust in the presence of outliers and heavy-tailed distributions. This improvement enables Trans-PtLR to more effectively capture true information in complex biological data, thereby enhancing estimation and prediction performance. By providing more accurate gene expression predictions, it aids in the understanding of gene regulatory mechanisms, supporting the advancement of personalized medicine. Furthermore, although the motivation for our research is gene expression prediction, the flexibility and robustness of the proposed framework for robust transfer learning make it potentially applicable to other fields, such as medical image analysis.

However, our method also has some limitations. First, the advantages of this method may be more pronounced under specific conditions, such as in datasets exhibiting skewed or heavy-tailed distributions, as demonstrated by our simulation studies. In contrast, the Trans-PtLR method does not show better performance than the Trans-PNLR method when errors follow a normal distribution. Secondly, our focus in this study is only on linear regression with *t*-distributed error. How to extend the robust transfer learning framework to other models such as Cox model and quantile regression to apply in the integration of multi-omics data is also an interesting problem. Thirdly, our methods used *l*_1_-penalty to control model sparsity and information level transferred from source data. It may be extended to *l*_2_-penalty or elastic net type of penalties, depending on the characteristics of the data at hand and whether the difference of regression parameters between a source and the target data is sparse or nearly sparse [35]. We can further consider different penalties for model sparsity control and transferred information level control. In addition, a prerequisite of our method is that it requires the same predictor set to be available in all sources, which may not be satisfied in real applications. How to deal with missing features appropriately while providing effective information for transfer is a direction that can be considered in the future.

In conclusion, Trans-PtLR is a robust transfer learning approach that can transfer valuable information from multiple source datasets to improve the performance of estimation and prediction in the target dataset under high dimensional scenarios. It offers robustness and flexibility to accommodate complex data with outliers and heavy tails.

## Supporting information

S1 Table The list of 54 tissues in GTEx dataset corresponding to their sample sizes. (DOCX)

S2 Table The accuracy of the transferable source detection algorithm in correctly identifying transferable datasets. (DOCX)

S3 Table The transferable sources detected by the proposed method Trans-PtLR and sample size of all transferable sources. (DOCX)

S4 Table The transferable sources detected by the method Trans-PNLR and sample size of all transferable sources. (DOCX)

S1 FigEstimation error ${\left\|{\hat{\beta }}^{\left(0\right)}-\beta^{\left(0\right)}\right\|}_{2}^{2}$ of candidate methods with a threshold t of 0.05.(TIF)

S2 FigEstimation error ${\left\|{\hat{\beta }}^{\left(0\right)}-\beta^{\left(0\right)}\right\|}_{2}^{2}$ of candidate methods with a threshold t of 0.15.(TIF)

S3 FigRelative prediction error of candidate methods relative to the PNLR with a threshold t of 0.05.We randomly split the target dataset into five folds, using four folds to train and the remaining fold to calculate the mean prediction error ${\left({\hat{Y}}_{i}-Y_{i}\right)}^{2}$ with ${\hat{Y}}_{i}=X_{i}^{T\left(0\right)}{\hat{\beta }}^{\left(0\right)}$.(TIF)

S4 FigRelative prediction error of candidate methods relative to the PNLR with a threshold t of 0.15.We randomly split the target dataset into five folds, using four folds to train and the remaining fold to calculate the mean prediction error ${\left({\hat{Y}}_{i}-Y_{i}\right)}^{2}$ with ${\hat{Y}}_{i}=X_{i}^{T\left(0\right)}{\hat{\beta }}^{\left(0\right)}$.(TIF)

S5 FigEstimation error ${\left\|{\hat{\beta_{1}}}^{\left(0\right)}-{\beta_{1}}^{\left(0\right)}\right\|}_{2}^{2}$ of candidate methods with a threshold t of 0.1.(TIF)

S6 FigEstimation error ${\left\|{\hat{\beta_{20}}}^{\left(0\right)}-{\beta_{20}}^{\left(0\right)}\right\|}_{2}^{2}$ of candidate methods with a threshold t of 0.1.(TIF)

S7 FigEstimation error ${\left\|{\hat{\beta }}^{\left(0\right)}-\beta^{\left(0\right)}\right\|}_{2}^{2}$ of candidate methods with a threshold t of 0.1, showing how estimation error varies with sample size.(TIF)

S1 Code File includes code "Transfer learning.R" and "updatebeta.cpp" for implementing transfer learning methods. (ZIP)
