# Supplementary material for: A robust transfer learning approach for high-dimensional linear regression to support integration of multi-source gene expression data
Source: PLoS Comput Biol. 2025 Jan 10;21(1):e1012739. doi: 10.1371/journal.pcbi.1012739 (PMC11756795; doi:10.1371/journal.pcbi.1012739)
Supplement: S1 Table — (DOCX) [file pcbi.1012739.s001.docx]

**S1 Table** The list of 54 tissues in GTEx dataset corresponding to their sample sizes.

| no. | Tissue | Sample Size |
| --- | --- | --- |
| 1 | Brain amygdala | 152 |
| 2 | Brain anterior cingulate cortex BA24 | 176 |
| 3 | Brain caudate basal ganglia | 246 |
| 4 | Brain cerebellar hemisphere | 215 |
| 5 | Brain cerebellum | 241 |
| 6 | Brain cortex | 255 |
| 7 | Brain frontal cortex BA9 | 209 |
| 8 | Brain hippocampus | 197 |
| 9 | Brain hypothalamus | 202 |
| 10 | Brain nucleus accumbens basal ganglia | 246 |
| 11 | Brain putamen basal ganglia | 205 |
| 12 | Brain spinal cord cervical | 159 |
| 13 | Brain substantia nigra | 139 |
| 14 | Adipose subcutaneous | 663 |
| 15 | Adipose visceral omentum | 541 |
| 16 | Adrenal gland | 258 |
| 17 | Artery aorta | 432 |
| 18 | Artery coronary | 240 |
| 19 | Artery tibial | 663 |
| 20 | Bladder | 21 |
| 21 | Breast mammary tissue | 459 |
| 22 | Cells cultured fibroblasts | 504 |
| 23 | Cells ebv-transformed lymphocytes | 174 |
| 24 | Cervix ectocervix | 9 |
| 25 | Cervix endocervix | 10 |
| 26 | Colon sigmoid | 373 |
| 27 | Colon transverse | 406 |
| 28 | Esophagus gastroesophageal junction | 375 |
| 29 | Esophagus mucosa | 555 |
| 30 | Esophagus muscularis | 515 |
| 31 | Fallopian tube | 9 |
| 32 | Heart atrial appendage | 429 |
| 33 | Heart left ventricle | 432 |
| 34 | Kidney cortex | 85 |
| 35 | Kidney medulla | 4 |
| 36 | Liver | 226 |
| 37 | Lung | 578 |
| 38 | Minor salivary gland | 162 |
| 39 | Muscle skeletal | 803 |
| 40 | Nerve tibial | 619 |
| 41 | Ovary | 180 |
| 42 | Pancreas | 328 |
| 43 | Pituitary | 283 |
| 44 | Prostate | 245 |
| 45 | Skin not sun exposed suprapubic | 604 |
| 46 | Skin sun exposed lower leg | 701 |
| 47 | Small intestine terminal ileum | 187 |
| 48 | Spleen | 241 |
| 49 | Stomach | 359 |
| 50 | Testis | 361 |
| 51 | Thyroid | 653 |
| 52 | Uterus | 142 |
| 53 | Vagina | 156 |
| 54 | Whole blood | 755 |
