# Supplementary material for: A robust transfer learning approach for high-dimensional linear regression to support integration of multi-source gene expression data
Source: PLoS Comput Biol. 2025 Jan 10;21(1):e1012739. doi: 10.1371/journal.pcbi.1012739 (PMC11756795; doi:10.1371/journal.pcbi.1012739)
Supplement: S2 Table — (DOCX) [file pcbi.1012739.s002.docx]

**S2 Table** The accuracy of the transferable source detection algorithm in correctly identifying transferable datasets.

|  |  |  | **Trans-PNLR**  **t error** | | | | **Trans-PtLR**  **CN error** | | | |
| --- | --- | --- | --- | --- | --- | --- | --- | --- | --- | --- |
| $\mathcal{t}$ | **h** | $\left\vert\mathcal{T} \right\vert$ | **N error** | $\boldsymbol{t}$ **error** | **St error** | **CN error** | **N error** | $\boldsymbol{t}$ **error** | **St error** | **CN error** |
| 0.05 | 10 | 0 | 99.4 | 97.16 | 96.84 | 98.12 | 99.5 | 96.83 | 97.25 | 98.84 |
|  |  | 2 | 99.8 | 96.43 | 95.95 | 98.89 | 99.3 | 97.93 | 97.68 | 99.36 |
|  |  | 4 | 99.5 | 95.22 | 95.6 | 98.37 | 99.4 | 97.33 | 97.08 | 98.38 |
|  |  | 6 | 99.2 | 95.71 | 95.51 | 98.5 | 99.39 | 98.21 | 96.84 | 99 |
|  |  | 8 | 99.9 | 95.1 | 96.11 | 98.9 | 99.8 | 98 | 96.77 | 99.2 |
|  |  | 10 | 100 | 96.5 | 96.1 | 99.6 | 99.7 | 98.5 | 99.4 | 99.9 |
|  | 20 | 0 | 100 | 98.65 | 98.54 | 100 | 100 | 98.9 | 99.28 | 100 |
|  |  | 2 | 98.3 | 96.91 | 97.19 | 98.7 | 99.6 | 98.18 | 98.9 | 99.1 |
|  |  | 4 | 97.5 | 97.73 | 97.53 | 98.5 | 99.3 | 98 | 98.69 | 98.9 |
|  |  | 6 | 96.4 | 96.06 | 95.57 | 97.7 | 98.5 | 97.8 | 96.9 | 98.1 |
|  |  | 8 | 94.5 | 95.7 | 96.1 | 96.7 | 99 | 97.5 | 98.3 | 97.9 |
|  |  | 10 | 92.8 | 94.4 | 94.4 | 95.9 | 98.1 | 95.4 | 96.7 | 98.1 |
| 0.1 | 10 | 0 | 99.5 | 96.67 | 96.03 | 98.23 | 99.1 | 97.72 | 97.12 | 98.65 |
|  |  | 2 | 99.7 | 95 | 95 | 98.32 | 99.6 | 97.18 | 97.44 | 98.98 |
|  |  | 4 | 99.8 | 96.32 | 93.98 | 99.09 | 99.39 | 97.5 | 97.41 | 99.08 |
|  |  | 6 | 99.8 | 96.85 | 96.15 | 99.39 | 99.7 | 97.31 | 97.61 | 99.49 |
|  |  | 8 | 99.8 | 96.02 | 94.64 | 99.5 | 99.5 | 97.35 | 98.35 | 99.4 |
|  |  | 10 | 99.9 | 96.8 | 97.3 | 99 | 99.9 | 98.5 | 98 | 99.9 |
|  | 20 | 0 | 100 | 98.35 | 98.6 | 100 | 100 | 99.28 | 99.39 | 100 |
|  |  | 2 | 99 | 98.74 | 97.01 | 99.2 | 99.4 | 99 | 99.3 | 99.7 |
|  |  | 4 | 97.6 | 96.43 | 96.74 | 98.8 | 99.8 | 97.7 | 98.5 | 99 |
|  |  | 6 | 97.5 | 94.9 | 96 | 97.6 | 98.9 | 97.7 | 98.7 | 98.2 |
|  |  | 8 | 94.2 | 96 | 96.5 | 97 | 98.9 | 95.7 | 95.8 | 97.7 |
|  |  | 10 | 90.1 | 95.5 | 95.3 | 97.2 | 97.3 | 95.7 | 96.7 | 98 |
| 0.15 | 10 | 0 | 99.8 | 96.03 | 96.43 | 98.42 | 99.1 | 97.47 | 97.68 | 98.65 |
|  |  | 2 | 99.6 | 96.62 | 95.66 | 98.54 | 99 | 96.28 | 96.74 | 99.26 |
|  |  | 4 | 99.39 | 95.56 | 95.57 | 99.4 | 99.08 | 97.07 | 97.19 | 98.89 |
|  |  | 6 | 99.8 | 96.11 | 96.78 | 99.09 | 99.39 | 96.67 | 97.28 | 99.39 |
|  |  | 8 | 100 | 95.71 | 92.96 | 98.48 | 99.8 | 96.43 | 96.97 | 99.6 |
|  |  | 10 | 100 | 96.4 | 96.1 | 99.2 | 99.7 | 99.3 | 99.1 | 99.9 |
|  | 20 | 0 | 100 | 98.62 | 99.14 | 100 | 100 | 99.79 | 99.7 | 100 |
|  |  | 2 | 98.8 | 98.84 | 98.44 | 99.1 | 99.8 | 98.67 | 98.14 | 99.6 |
|  |  | 4 | 98.2 | 96.7 | 96.49 | 98.1 | 99.3 | 98.16 | 97.68 | 99 |
|  |  | 6 | 96.6 | 96.29 | 96.87 | 97.9 | 99.4 | 98.08 | 97.24 | 98.1 |
|  |  | 8 | 95.6 | 96.8 | 95.1 | 97.4 | 98.6 | 97.5 | 95.8 | 98.3 |
|  |  | 10 | 92.9 | 94.9 | 95.2 | 96.7 | 97.3 | 96.9 | 96.2 | 98 |
