# Supplementary material for: A robust transfer learning approach for high-dimensional linear regression to support integration of multi-source gene expression data
Source: PLoS Comput Biol. 2025 Jan 10;21(1):e1012739. doi: 10.1371/journal.pcbi.1012739 (PMC11756795; doi:10.1371/journal.pcbi.1012739)
Supplement: S4 Table — (DOCX) [file pcbi.1012739.s004.docx]

**Table S4** The transferable sources detected by the method Trans-PNLR and sample size of all transferable sources.

|  | T1 | T2 | T3 | T4 | T5 | T6 | T7 | T8 | T9 | T10 | T11 | T12 | T13 |
| --- | --- | --- | --- | --- | --- | --- | --- | --- | --- | --- | --- | --- | --- |
| Adipose Subcutaneous | × | × | √ | √ | × | × | × | × | √ | × | √ | √ | √ |
| Adipose Visceral  omentum | √ | × | × | √ | × | × | √ | × | × | × | √ | √ | × |
| Adrenal gland | × | × | × | × | × | × | √ | √ | × | × | √ | √ | × |
| Artery Aorta | √ | √ | √ | √ | × | × | × | × | × | × | × | √ | × |
| Artery Coronary | √ | × | √ | √ | × | × | × | √ | √ | × | √ | √ | × |
| Artery Tibial | √ | √ | × | √ | × | × | × | × | × | × | × | √ | × |
| Breast Mammary tissue | √ | √ | × | √ | × | × | × | √ | × | × | × | √ | × |
| Cells Cultured fibroblasts | √ | × | √ | × | × | × | × | × | × | × | √ | √ | × |
| Ebv-transformed  lymphocytes | √ | × | √ | × | × | × | √ | × | × | × | √ | × | × |
| Colon Sigmoid | √ | × | √ | √ | × | × | × | × | × | √ | √ | √ | × |
| Colon Transverse | × | × | √ | √ | √ | √ | √ | √ | × | √ | √ | √ | × |
| Esophagus Gastroesophageal junction | √ | × | √ | × | × | × | √ | × | × | × | √ | × | × |
| Esophagus Mucosa | √ | √ | × | √ | × | × | × | √ | √ | × | × | √ | × |
| Esophagus Muscularis | × | × | √ | × | × | √ | √ | √ | × | × | × | × | √ |
| Heart Atrial appendage | √ | √ | × | × | × | × | × | √ | × | × | × | × | × |
| Heart Left ventricle | √ | × | × | × | × | × | √ | × | × | × | × | √ | √ |
| Kidney Cortex | √ | × | √ | √ | √ | × | √ | × | × | √ | √ | √ | × |
| Liver | √ | √ | √ | × | × | × | √ | √ | √ | × | √ | √ | × |
| Lung | √ | √ | √ | × | × | × | × | × | √ | × | √ | √ | × |
| Minor salivary gland | √ | √ | √ | √ | × | × | √ | × | × | × | √ | √ | × |
| Muscle skeletal | × | √ | × | × | √ | × | √ | √ | × | × | × | √ | × |
| Nerve tibial | × | × | √ | × | × | × | √ | √ | × | × | × | √ | × |
| Ovary | √ | × | √ | √ | × | √ | √ | × | √ | × | √ | √ | × |
| Pancreas | √ | × | × | × | × | × | √ | × | × | × | √ | √ | × |
| Pituitary | √ | √ | √ | √ | √ | × | × | × | × | × | √ | √ | × |
| Prostate | √ | × | × | × | × | × | × | × | × | × | √ | √ | × |
| Skin Not sun exposed  suprapubic | × | × | √ | √ | × | × | × | × | × | × | √ | √ | × |
| Skin Sun exposed  lower leg | × | × | √ | √ | × | × | × | × | × | × | √ | √ | × |
| Small intestine terminal  ileum | × | × | × | √ | × | × | √ | × | √ | × | √ | √ | × |
| Spleen | √ | × | √ | × | × | √ | × | × | √ | × | √ | × | × |
| Stomach | √ | √ | × | √ | × | × | √ | √ | √ | × | √ | √ | × |
| Testis | √ | × | √ | √ | √ | √ | × | √ | × | × | × | √ | × |
| Thyroid | √ | √ | √ | × | × | × | × | √ | √ | √ | √ | √ | × |
| Uterus | √ | × | × | √ | × | √ | × | √ | × | √ | √ | √ | × |
| Vagina | √ | √ | √ | √ | × | √ | × | √ | √ | × | × | √ | × |
| Whole blood | √ | × | √ | √ | × | × | √ | × | × | × | √ | × | × |
| Sample Size | 9931 | 5758 | 9286 | 8307 | 1938 | 2001 | 6405 | 6181 | 4038 | 1659 | 9263 | 12198 | 1610 |

T1-T13 are 13 targets: Brain Amygdala, Brain Anterior cingulate cortex BA24, Brain caudate basal ganglia, Brain Cerebellar hemisphere, Brain Cerebellum, Brain Cortex, Brain Frontal cortex BA9, Brain Hippocampus, Brain Hypothalamus, Brain Nucleus accumbens basal ganglia, Brain Putamen basal ganglia, Brain Spinal cord cervical, and Brain Substantia nigra.
