# Supplementary figures and images for: A robust transfer learning approach for high-dimensional linear regression to support integration of multi-source gene expression data
Source: PLoS Comput Biol. 2025 Jan 10;21(1):e1012739. doi: 10.1371/journal.pcbi.1012739 (PMC11756795; doi:10.1371/journal.pcbi.1012739)

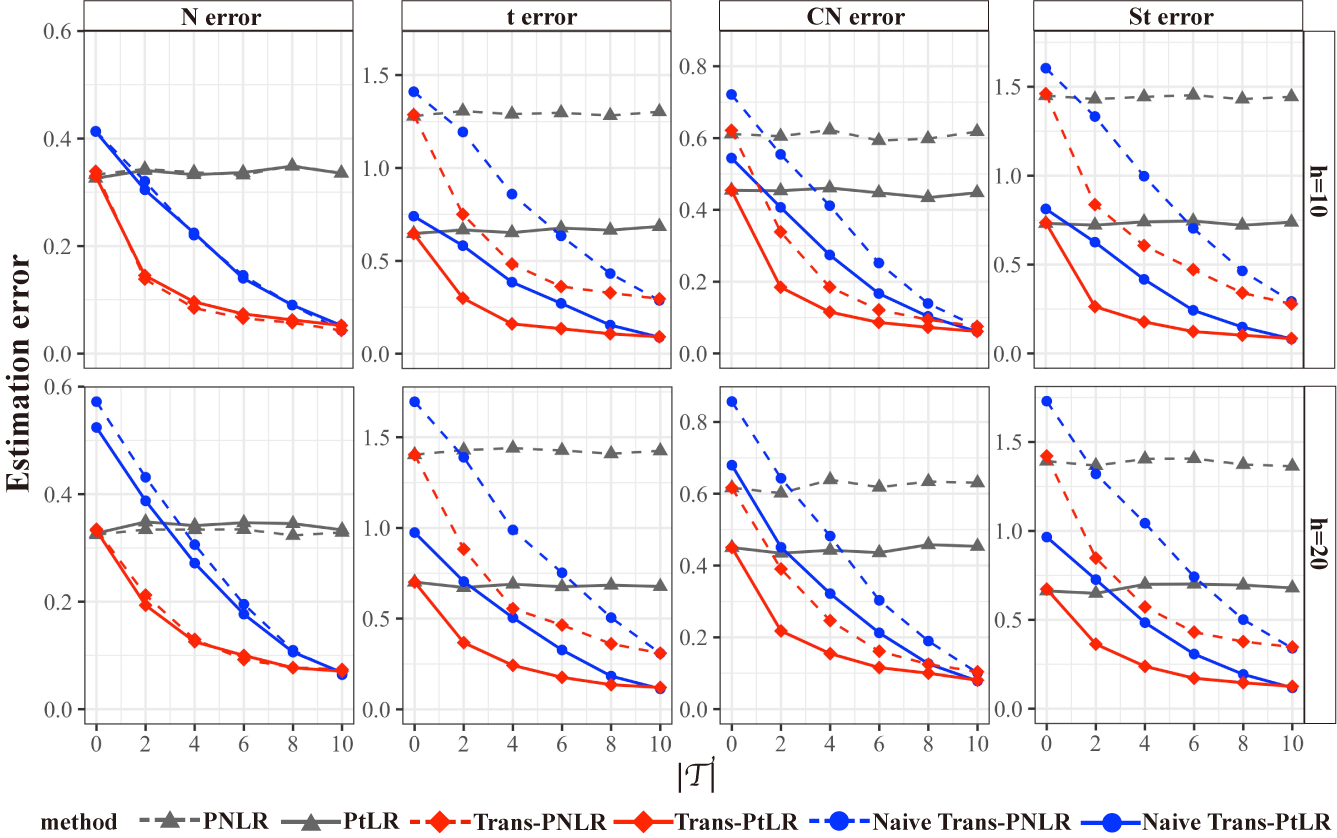

Supplement: S1 Fig — (TIF) [file pcbi.1012739.s005.tif]

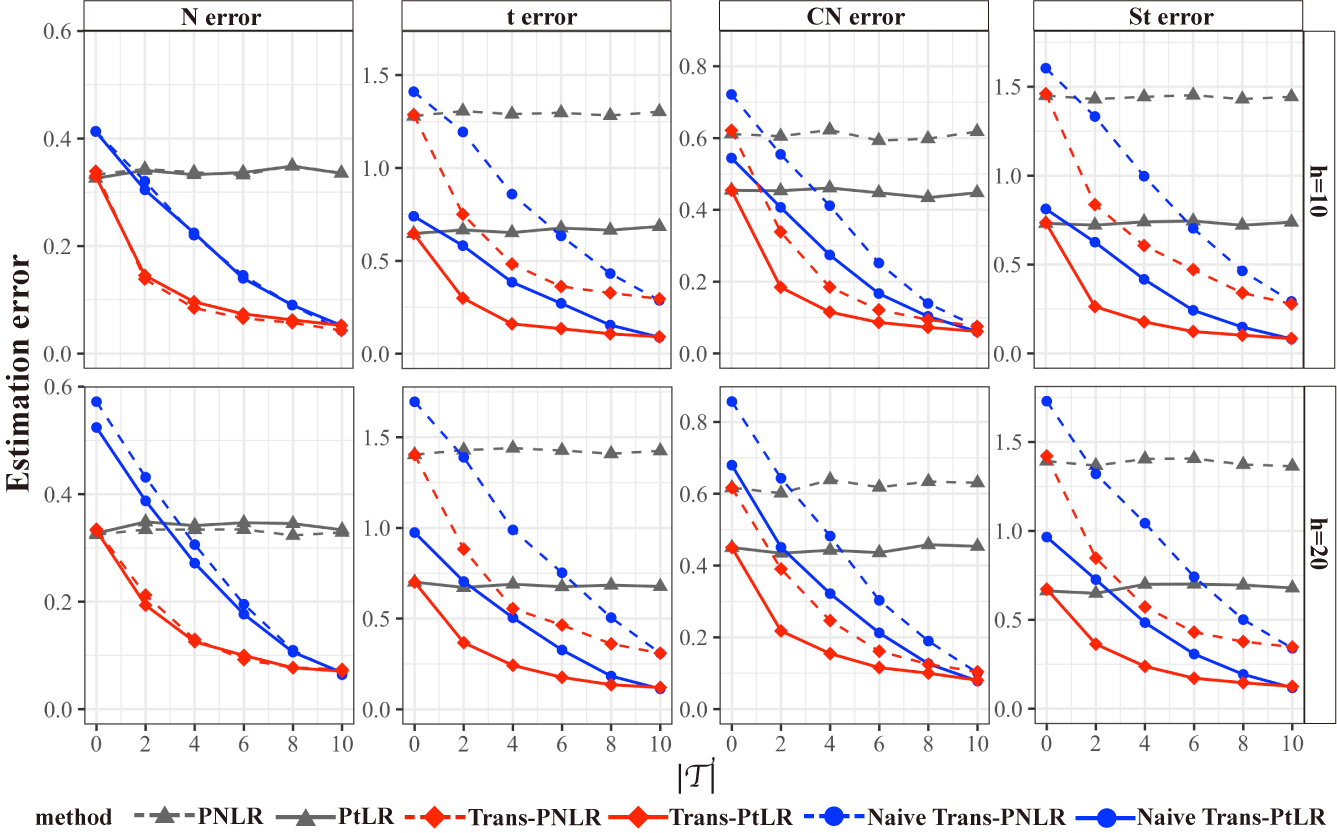

Supplement: S2 Fig — (TIF) [file pcbi.1012739.s006.tif]

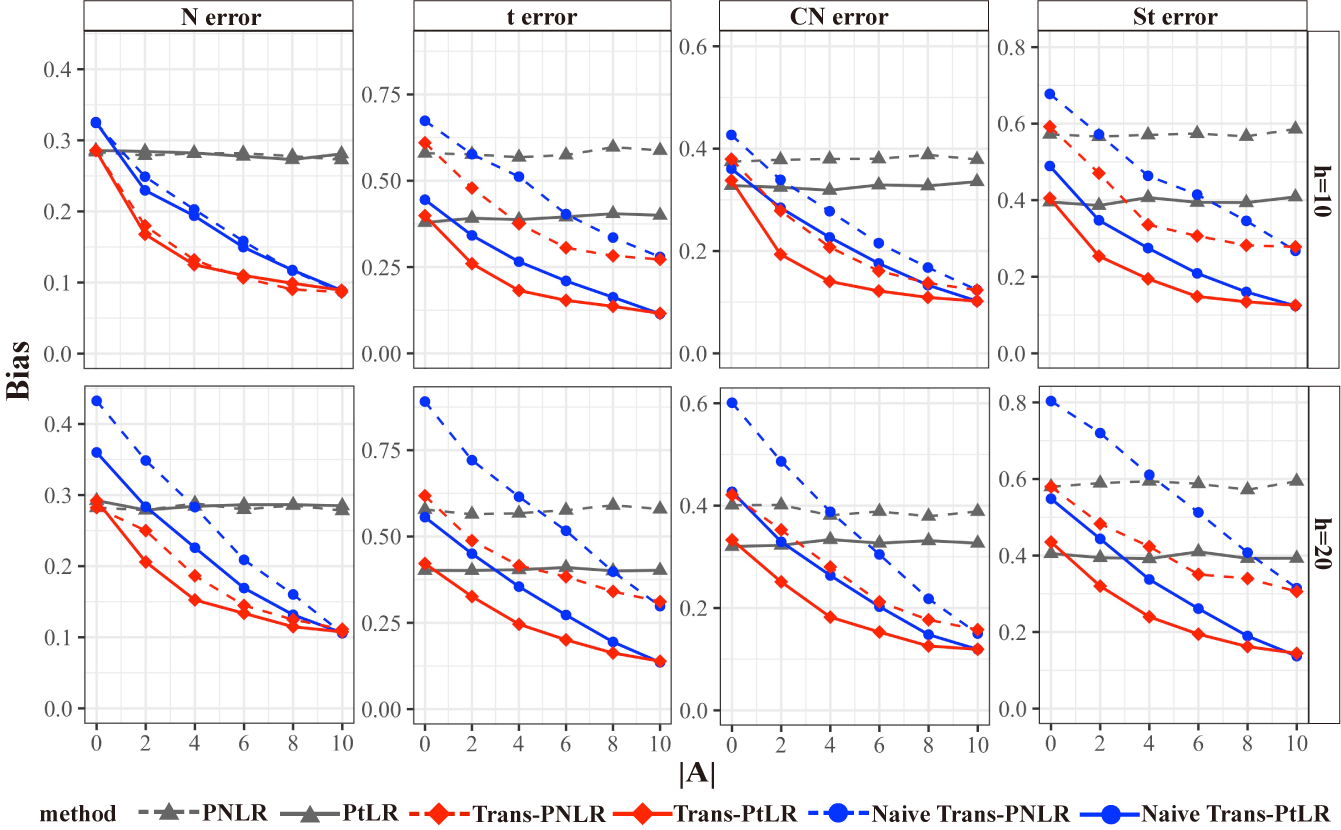

Supplement: S3 Fig — We randomly split the target dataset into five folds, using four folds to train and the remaining fold to calculate the mean prediction error Y^i-Yi2 with Y^i=XiT0β^0. (TIF) [file pcbi.1012739.s007.tif]

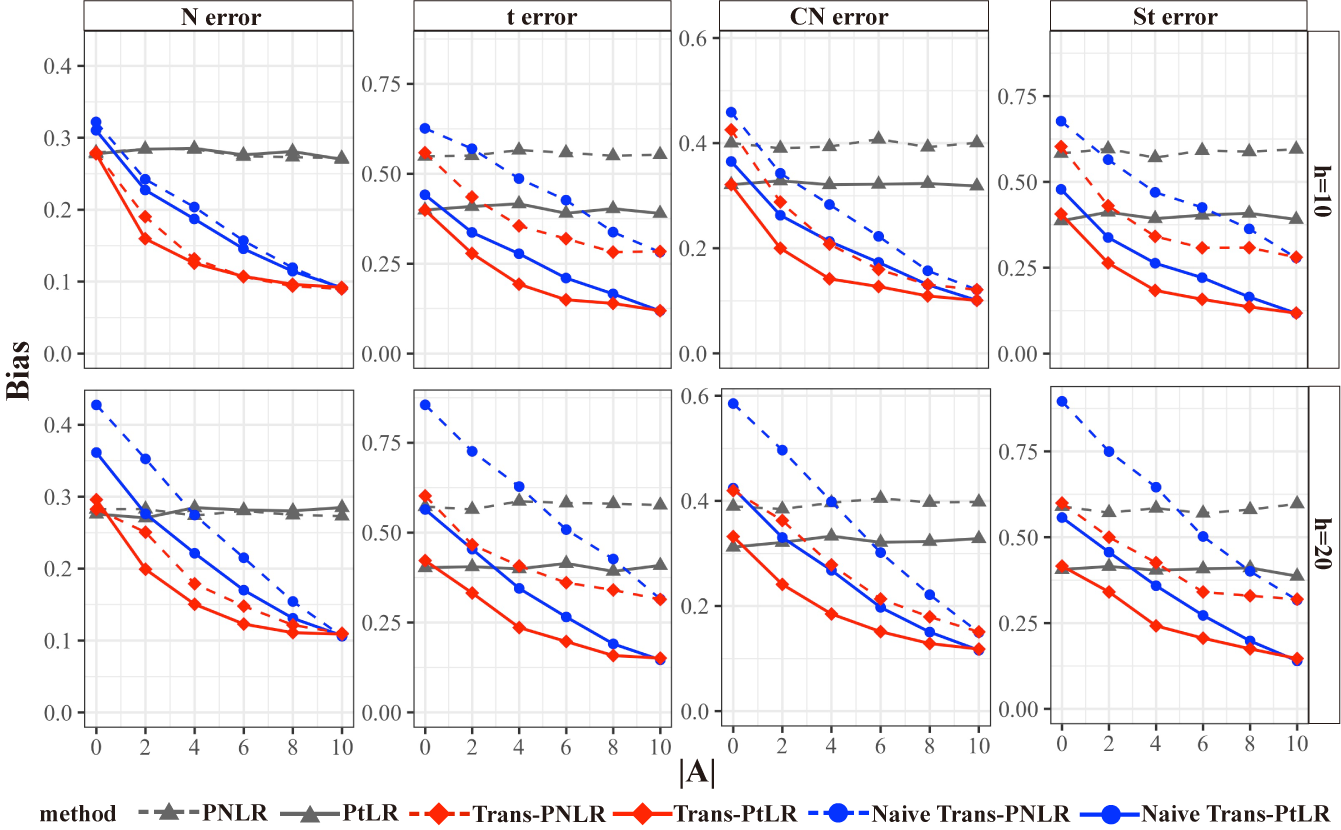

Supplement: S4 Fig — We randomly split the target dataset into five folds, using four folds to train and the remaining fold to calculate the mean prediction error Y^i-Yi2 with Y^i=XiT0β^0. (TIF) [file pcbi.1012739.s008.tif]

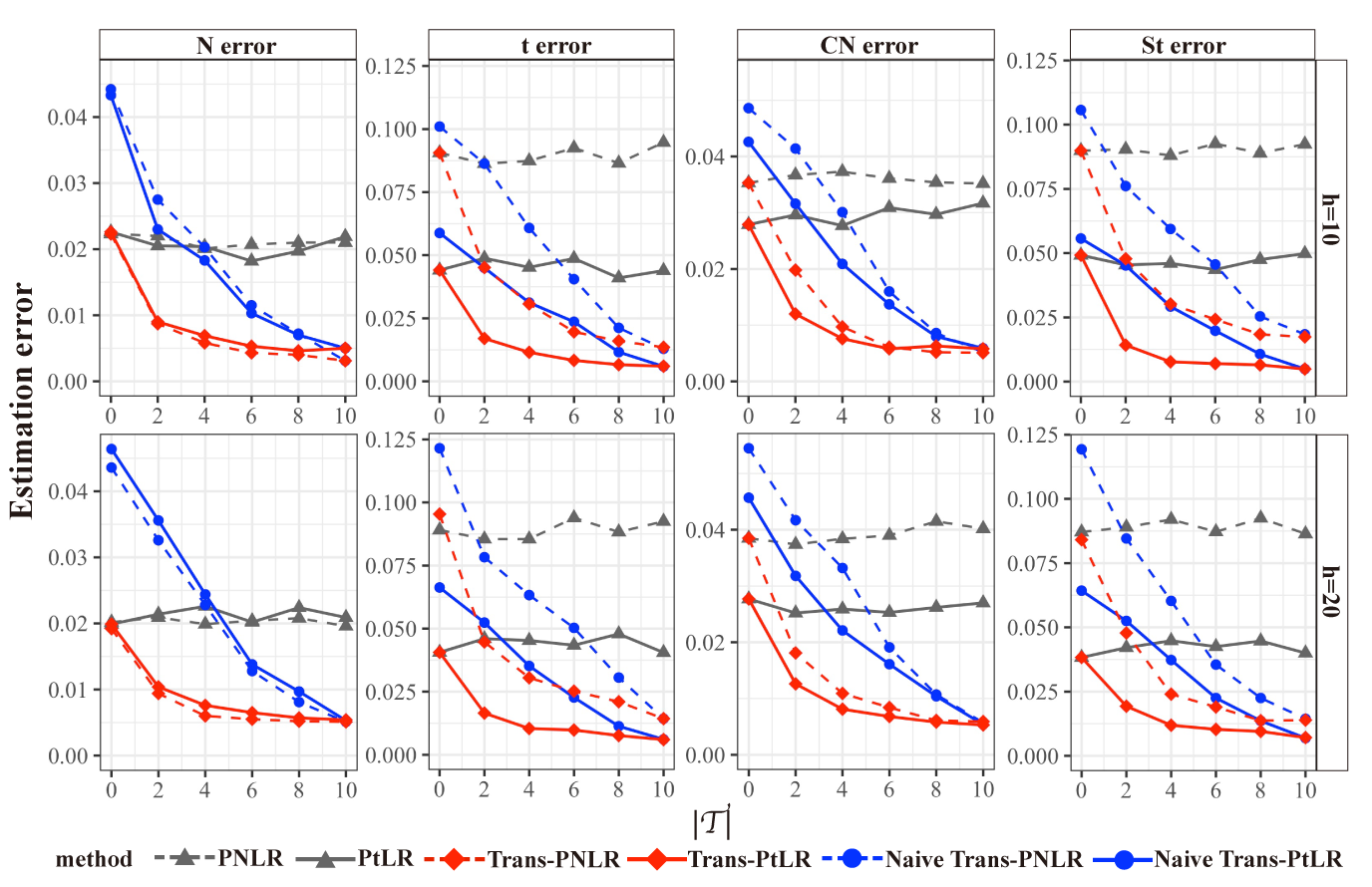

Supplement: S5 Fig — (TIF) [file pcbi.1012739.s009.tif]

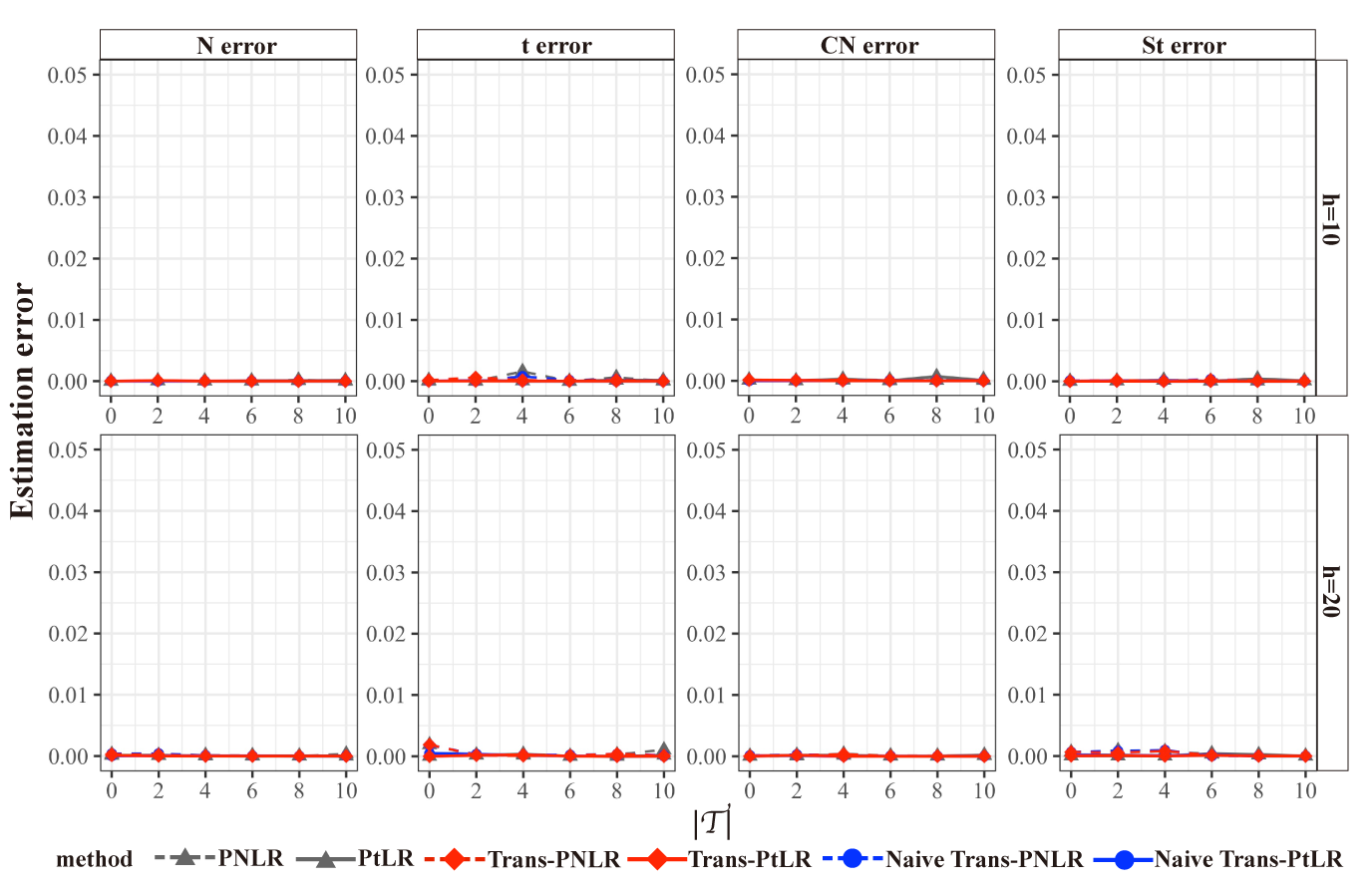

Supplement: S6 Fig — (TIF) [file pcbi.1012739.s010.tif]

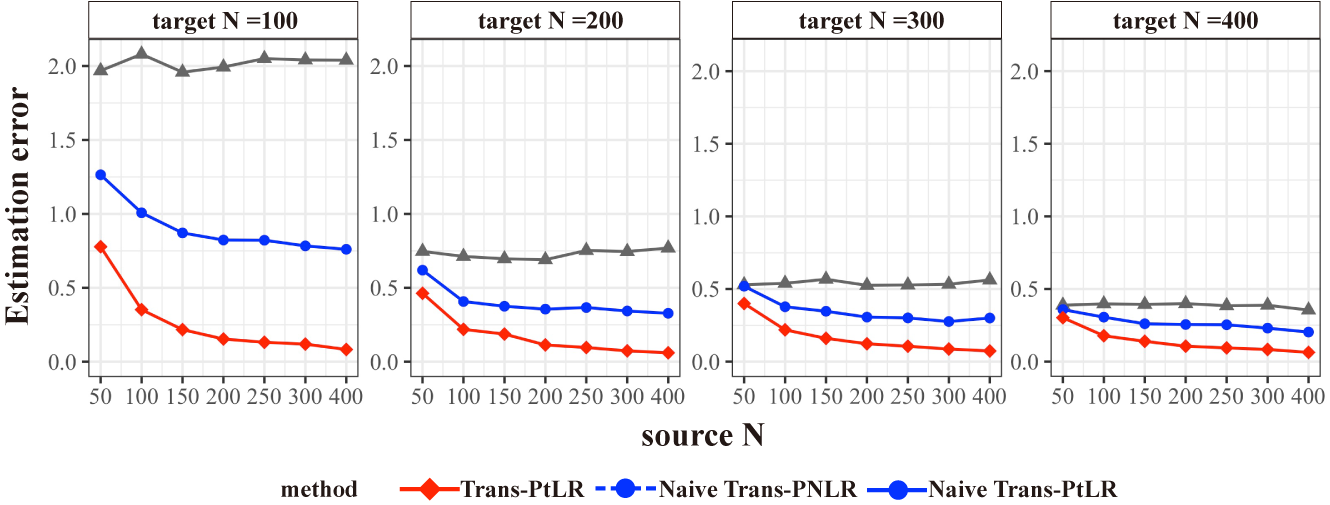

Supplement: S7 Fig — (TIF) [file pcbi.1012739.s011.tif]
